# Sialylated glycans as receptor and inhibitor of enterovirus 71 infection to DLD-1 intestinal cells

**DOI:** 10.1186/1743-422X-6-141

**Published:** 2009-09-15

**Authors:** Betsy Yang, Hau Chuang, Kuender D Yang

**Affiliations:** 1 Kaohsiung American School and Department of Medical Research, Chang Gung Memorial Hospital-Kaohsiung Medical Center, Chang Gung University, Kaohsiung 833, Taiwan, Republic of China

## Abstract

**Background:**

Many viruses recognize specific sugar residues, particularly sulfated or sialylated glycans, as the infection receptors. A change of sialic acid (2-6)-linked galactose (SA-α2,6Gal) to SA-α2,3Gal determines the receptor for avian flu infection. The receptor for enterovirus 71 (EV71) infection that frequently causes fatal encephalitis in Asian children remains unclear. Currently, there is no effective vaccine or anti-virus agent for EV71 infection. Using DLD-1 intestinal cells, this study investigated whether SA-linked glycan on DLD-1 intestinal cells was a receptor for EV71, and whether natural SA-linked sugars from human milk could block EV71 infection.

**Results:**

EV71 specifically infected DLD-1 intestinal cells but not K562 myeloid cells. Depletion of O-linked glycans or glycolipids, but not N-linked glycans, significantly decreased EV71 infection of DLD-1 cells. Pretreatment of DLD-1 cells with sialidase (10 mU, 2 hours) significantly reduced 20-fold EV71 replication (p < 0.01). Taken together, these results suggest that SA-linked O-glycans and glycolipids, but not N-glycans, on DLD-1 cells were responsible for EV71 infection. Purified SA-α2,3Gal and SA-α2,6Gal from human milk significantly inhibited EV71 infection of DLD-1 cells, indicating terminal SA-linked glycans could be receptors and inhibitors of EV71 infection.

**Conclusion:**

This is the first in the literature to demonstrate that EV71 uses SA-linked glycans as receptors for infection, and natural SA-linked glycans from human milk can protect intestinal cells from EV71 infection. Further studies will test how a SA-containing glycan can prevent EV71 in the future.

## Introduction

Many viruses recognize specific sugar residues, particularly sulfated or sialylated glycans, as the infection receptors. Avian influenza virus and human influenza virus use different sugar residues as their receptors, resulting in different host range of infections [1]. Enterovirus 71 (EV71) which prevails almost every summer season and causes hand-foot-mouth disease is frequently complicated with fatal encephalitis in Asia, and even Europe [2-6]. Currently, there is neither vaccine available for prevention of EV71, nor antiviral treatment for EV71 infection. Before development of effective antiviral agents or specific vaccine available to control epidemics of EV71, identification of the receptor(s) for EV71 and block of the receptor(s) may be a good regimen for prevention of EV71 infection.

Sialic acid (SA) also known as neuraminic acid is usually linked to galactose or other sugar residues as an antenna of blood group antigens, tumor antigens or viral receptors [7]. Gastrointestinal and respiratory epithelial cells expressed abundant SA-containing glycoproteins and SA-containing glycolipids [1,8,9]. It is known that sialic acid-α2,6 galactose (SA-α2,6Gal) epitope is a receptor for human influenza virus [1] and sialic acid-α2,3galactose (SA-α2,3Gal) is a receptor for coxackievirus A24 [8]. The transmission route of the EV71 is fecal-oral and/or droplet-aerosol route, and the receptor for EV71 is unknown [3]. We, therefore, postulated that EV71 might use the SA-linked glycan on intestinal epithelial cells as a receptor, and natural SA-linked glycans may prevent human intestinal cells from EV71 infection.

This study was conducted to investigate whether depletion of glycolipids, N-linked glycans or O-linked glycans on DLD-1 intestinal cells could avoid EV71 infection, and administration of SA-linked sugars from human milk could block EV71 infections. If natural SA-linked sugars could block EV71 infection, SA-linked glycans may be made to prevent EV71 infections.

## Results and Discussion

### EV71 infection of DLD-1 intestinal cells

**Figure 1:**
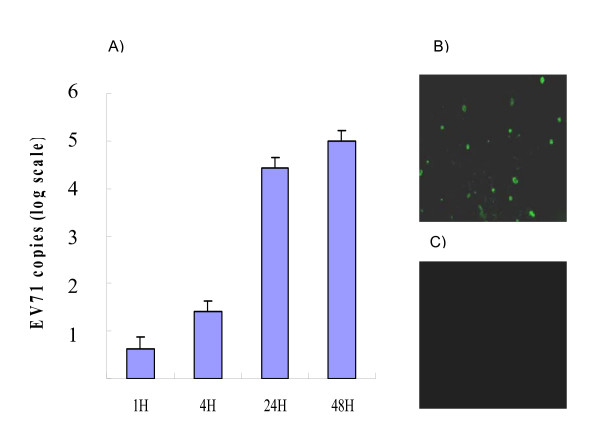
**Infection of DLD-1 cells by EV71**. EV71 infected and replicated in DLD-1 cells within 4 hours (4 H), and rapidly replicated in 24 hours (24 H) (A). The replication of EV71 in DLD-1 cells could be visible by specific antibody directed immunofluorescent staining (B). In contrast, EV71 did not infect K562 myeloid cells in 24 hours (C). Data presented are calculated from 4 experiments.

Experiments were initially performed to study whether EV71 could specifically infect DLD-1 intestinal cells. Using multiplication of index (MOI) of 10, it was found that EV71 could infect and replicate in DLD-1 cells in 4 hours, and caused a dramatic increase of replication in 24 hours of EV71 infection. As determined by RT-PCR analysis of the virus titers, the virus replication (RNA copies of EV71) increased from 67 copies of EV71 per ml in one hour to more than 10^6 ^copies/ml in 2 days (Figure 1A). As demonstrated by indirect immunofluorescent assay (IFA), EV71 replication was detectable in 24 hours of infection (Figure 1B). In contrast, EV71 did not infect K562 myeloid cells in 24 hours (Figure 1C). This result suggests that EV71 infection has tissue specificity. No detectable infection of EV71 on K562 myeloid cells by IFA might be due to lack of EV71 receptors on myeloid cells or limited replication of EV71 in K562 myeloid cells.

### SA-linked O-glycan and glycolipid but not N-glycan responsible for EV71 infection

**Figure 2:**
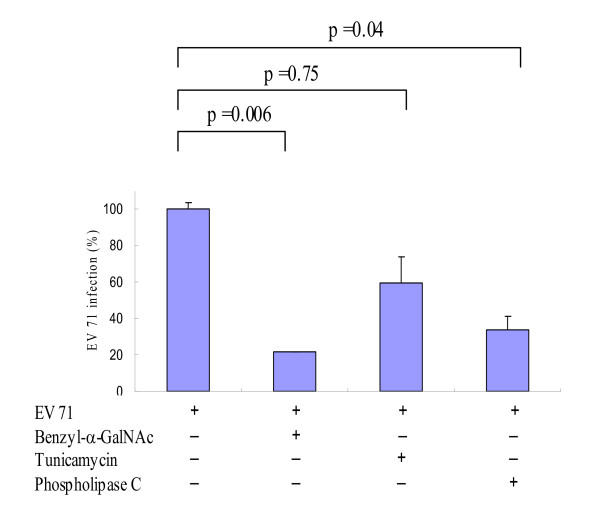
**O-glycans and glycolipids but not N-glycans responsible for the EV71 infection**. O-glycan synthesis inhibitor, benzyl N-acetyl-α-D-galactosaminide and glycolipid anchorage inhibitor, phosphotidylinositol-specific phospholipase, but not N-glycan synthesis inhibitor, tunicamycin, significantly inhibited EV71 infections. Data presented were calculated from three experiments.

To determine whether glycans were involved in the EV71 infection of intestinal cells, we tested whether depletion of N-glycans, O-glycans or glycolipids blocked EV71 infection of DLD-1 cells. We used benzyl N-acetyl-α-D-galactosaminide (3 mM), tunicamycin (0.2 mg/ml) and phosphotidylinositol-specific phospholipase (5 U/ml) to deplete O-linked glycans, N-linked glycans and glycolipids of DLD-1 cells, respectively. It was found that depletion of O-linked glycan or glycolipid, but not N-linked glycan, significantly decreased EV71 infection of DLD-1 cells (Figure 2). Particularly, O-linked glycan was the major entry of EV71 infection because depletion of O-glycans by benzyl N-acetyl-α-D-galactosaminide (3 mM) reduced the most EV71 infection (P = 0.006). This is compatible to a recent report demonstrating a sialomucin (O-linked glycoprotein) membrane protein (CD162) as a functional receptor for enterovirus 71 infection [10].

### Inhibition of EV71 infection in DLD-1 cells by sialidase treatment

**Figure 3:**
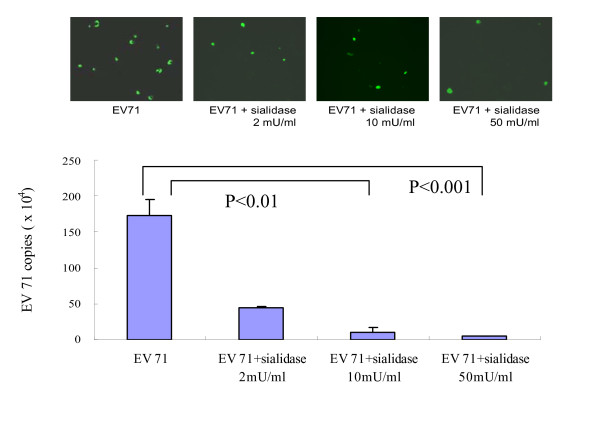
**Sialidase treatment of DLD-1 cells decreased EV71 infection**. DLD-1 cells pre-treated with different doses of sialidase for 2 hours significantly reduced EV71 infection in 24 hours under immunofluorescent assay (upper panel). The treatment of sialidase at 10 mU/ml (P < 0.01) or at 50 mU/ml (P < 0.001) significantly inhibited EV71 infection of DLD-1 cells for 3 days as analyzed by qRT-PCR analysis of EV71 titers (lower panel). Data presented are derived from 4 experiments.

Considering O-linked glycans and glycolipids usually have a variety of terminal sialic acid (SA) residues that may contribute to the binding of EV71, we depleted surface SA from SA-α2,3 Gal and SA-α2,6Gal of DLD-1 cells by preincubation of the α2,3 and α2,6 sialidase. The sialidase treatment at 2, 10, and 50 mU/ml for 2 hours significantly decreased EV71-infected cells of DLD-1 cells in immunoflueorescent staining (Figure 3, upper panel). Pretreatment of sialidase also significantly reduced the EV71 replication in DLD-1 cells for 3 days from 1.7 × 10^6 ^copies/ml down to 7.0 × 10^4 ^copies/ml, with more than 20-fold reduction (Figure 3, lower panel). This experiment suggests that SA-linked glycans on intestinal cells are responsible for the entry of EV71 infection. Pretreatment of sialidase directed against SA-α2,3Gal and SA-α2,6Gal significantly (P < 0.01) reduced EV71 infection suggesting sialylated galactose epitopes are responsible for EV71 infection of DLD-1 intestinal cells. Experiments were next performed to study whether natural SA-containing sugars such as SA-α2,3Gal and SA-α2,6Gal could block EV71 infection in DLD-1 intestinal cells below.

### Blockade of EV71 infection by SA-derived glycans from milk

**Figure 4:**
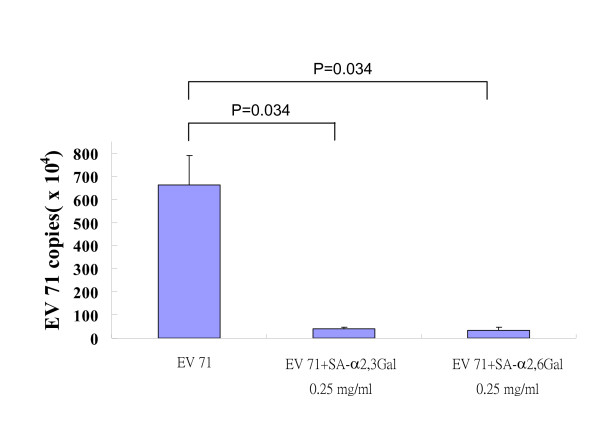
**Inhibition of EV71 infection in DLD-1 cells by SA-α2,3Gal and SA-α2,6Gal**. DLD-1 cells co-incubated with SA-linked galactose (SA-α2,3Gal or SA-α2,6Gal) significantly (P = 0.034) reduced the EV71 infection for 3 days as quantified by RT-PCR analysis. Data presented are calculated from 4 experiments.

Purified SA-α2,3Gal (molecular weight 633) and SA-α2,6Gal (molecular weight 655) from human milk (0.25 mg/ml) were used to inhibit EV71 infection of DLD-1 cells. As showed in Figure 4, incubation of SA-α2,3Gal and SA-α2,6Gal with EV71 before infection significantly (P = 0.034) inhibited EV71 infection of DLD-1 intestinal cells. Both SA-α2,3Gal and SA-α2,6Gal from human milk could inhibit EV71 infections, suggesting human breast feeding might prevent infants from EV71 infection via gastrointestinal tract.

## Materials and methods

Study design. This study initially studied whether EV71 specifically infected DLD-1 intestinal cells, but not K562 myeloid cells. Using DLD-1 intestinal cells which possess abundant sialylated glycans on cell surface, we tested whether depletion of glycolipids or glycoproteins (N-linked glycoprotein or O-linked glycoproteins) blocked EV71 infection, and sialidase depletion of SA residues on DLD-1 cells protected DLD-1 cells from EV71 infection, indicating SA-linked glycan responsible for the entry of EV71 infection. Finally, we used SA-linked glycans purified from human milk to block EV71 infection of DLD-1 cells in order to validate SA-linked galactose residues responsible for EV71 infection.

**Figure 5:**
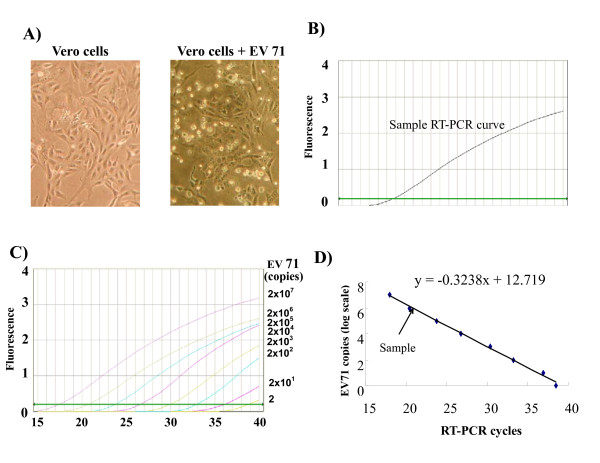
**Culture and quantification of EV71**. EV71 were cultured in Vero cells at MOI = 2 until visible cytopathic effect (CPE) (A), and subjected to qRT-PCR analysis of virus titer (B). Based on qRT-PCR detection of a series of well-known EV71 titers (C), the virus titers were determined by an interpolation on the standard curve (D).

Preparation of EV71. A clinical isolate of EV71 defined by EV71-specific antibody was obtained from the Laboratory of Virology, Department of Pathology, Chang Gung Memorial Hospital, Kaohsiung. EV71 were cultured and harvested from Vero cells. Vero cells at 5 × 10^5 ^cells/ml were cultured in 75 cm^2 ^culture flasks for over night, and inoculated with EV71 at multiplicity of infection (MOI) = 2 for 6 days. When more than 60% Vero cells revealed cytopathic effect (CPE), the total cell pellet was set to freeze and thaw for 3 times before the virus harvested by centrifugation at 1500 g for 10 minutes to separate viruses from cell debris. The virus titer was adjusted to 2 × 10^7 ^copies/ml based on RT-PCR quantification of EV71 virus copies (Figure 5), and stored in aliquots at -80°C before studies.

Culture of human intestine epithelial cell line. We used DLD-1 intestinal epithelial cells as a target of EV71 infection, and K562 myeloid leukemia cells were used as control cells for comparison. The DLD-1 and K562 cell lines were obtained from Food Industrial Research Institute, Hsin-chu, Taiwan. The reason to use intestinal cell line is because it can express different levels of SA-linked glycans resembling neonatal rat intestine [9]. DLD-1 cells were cultured in Roswell Park Memorial Institute 1640 (RPMI1640) medium with 10% fetal bovine serum, and harvested into 2 × 10^6 ^cells/ml for testing whether depletion of SA-linked glycans on DLD-1 cells by sialidase treatment reduced EV71 infection. Experiments were also performed to differentiate whether different natural SA-linked glycans such as SA-α2,3Gal or SA-α2,6Gal could protect against EV71 infections.

Determination of EV71 infection by indirect immunofluorescence and RT-PCR analysis. Infection of EV71 was assessed by an indirect immunofluorescent staining with an EV71-specific monoclonal antibody (Chemicon Inc. CA). DLD-1 cells (2 × 10^5 ^cells/ml) with and without sialidase pre-treatment for 2 hours were subjected to EV71 infection. The EV71 infected DLD-1 cells were harvested in one day for staining with mouse anti-EV71 monoclonal antibody or nonspecific antibody after cold acetone fixation, followed by FITC-labeled goat anti-mouse immunoglobulin antibody for fluorescent visualization. Cells were also harvested in 3 days for quantification of EV71 replication by a real time quantitative RT-PCR (qRT-PCR) analysis as previously described [11]. The primers used to detect EV71 RNA copies by SYBR Green fluorescent RT-PCR were forward: 5'-CCCCTGAATGCGGCTAATC-3' and reverse: 5'-CCATATAGCTATTGGATTGGCCA-3'. The copies of virus titers were calculated based on a standard curve made by a series of well-known RNA copies of EV71.

Differentiation of the SA-containing glycoproteins and glycolipids responsible for EV71 infection. Sugar residues on cell surface are usually linked to protein, called glycoprotein, or linked to lipid, called glycolipid. Employing inhibitors of protein glycosylation and lipid glycosylation synthesis, we investigated if the SA-based residue responsible for EV71 infection stems from glycoprotein or glycolipid. To test whether N-linked or O-linked sialylglycoprotein on DLD-1 cells was the receptor for EV71, DLD-1 cells (1 × 10^6 ^cells/ml) were respectively incubated with 3 mM benzyl N-acetyl-α-D-galactosaminide (Sigma-Aldrich Inc., St. Louis, MO) for 48 hours or with 0.2 mg/ml tunicamycin (Sigma-Aldrich Inc.) for 24 hours before subjected to EV71 binding assay at MOI = 10. To test whether sialylglycolipid on DLD-1 cells was the receptor for EV71, DLD-1 cells (1 × 10^6 ^cells/50 μl) were incubated with 50 μl phosphotidylinositol-specific phospholipase (5 U/ml) purchased from Sigma-Aldrich Inc. for 90 minutes before subjected to the test of EV71 binding assay. The EV71 binding assay was performed within one hour after washing out the treatment of specific inhibitor because DLD-1 cells in the inhibitor-free condition could re-express glycoprotein or glycolipid that might interfere the experimental interpretation.

Sources of sialidase and natural SA-linked glycans: The sialidase (α2-3, 6-sialidase, *Clostridium perfringens*) that can cleave SA from SA-α2,3Gal and/or SA-α2,6Gal compounds was purchased from Calbiochem Inc., Darmstadt, Germany. For experiments, DLD-1 cells were pretreated with 2, 10 or 50 mU/ml sialidase for 2 hours before subjected to EV71 infections for 24 hours at MOI = 10. Human milk SA-α2,3Gal (97% purity) and SA-α2,6Gal (98% purity) were purchased from Sigma-Aldrich Inc. (St. Louis, MO) and tested for inhibition of EV71 infections.

Determination of SA-linked sugar residue as a receptor and inhibitor of EV 71 infection by qRT-PCR. To validate whether SA-linked sugar residues were receptors for EV71 infection, EV71 (2 × 10^6 ^copies/ml) were co-incubated with SA-α2,3Gal (0.25 mg/ml) or SA-α2,6Gal (0.25 mg/ml) 15 minutes before added to infect DLD-1 cells (2 × 10^5 ^cells/ml) for 3 days. Replication of EV71 in DLD-1 cells was determined by qRT-PCR detection as described above.

Data management and statistics. Specific infection of EV71 to DLD-1 cells was compared to the control cell line, K562, myeloid leukemia cells. Binding of EV71 to SA-linked sugars was validated by depletion of SA by sialidase treatment. Inhibition of EV71 by different SA-linked sugar residues was analyzed by non-parametric analysis of Mann-Whitney U test. Chemical structures of the SA-linked compounds were drawn by the software of Chemwindow 6.0 (Bio-Rad Inc., Hercules, CA).

## Conclusion

**Figure 6:**
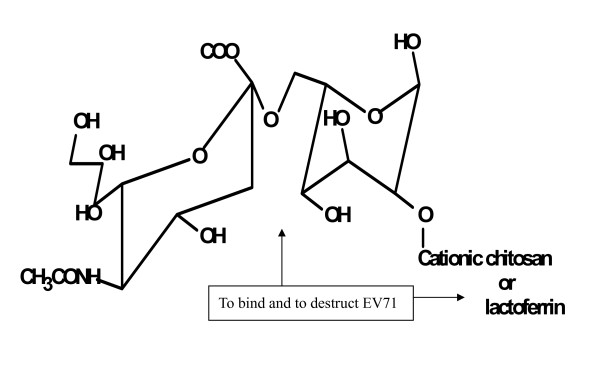
**A proposed SA-based "double-edge sword" on blocking and destructioing of EV71 infections**. SA (α2,6)-linked galactose can block EV71 infection by competition of sugar receptor, and the galactose can be linked with cationic compounds such as lactoferrin or chitosan for destruction of EV71.

This is the first in the literature to demonstrate that both SA-α2,6Gal and SA-α2,3Gal are responsible for EV71 infection of DLD-1 intestinal cells. Interruption of sugar-lectin interactions for antiviral treatment has been recently described [12]. Natural SA-containing glycans in human milk could inhibit EV71 infections, suggesting that human breast feeding may prevent infants from EV71 infection. Based on a strategy to link a SA-derived sugar residue which can compete EV71 invasion receptor to an antiviral agent such as cationic protein, lactoferrin, which can direct against EV71 by targeting viral envelope [13], or other cationic compounds such as chitosan that possesses not only cationic charge but also feasibility of making nanoparticles, we may be able to use the SA-linked antiviral agent as a "double-edge sword" to bind EV71 and destruct EV71 simultaneously, as shown in Figure 6. Interpretation of this *in vitro *study is limited by the lack of infectivity data and lack of data from animal model to support the infection inhibition by IFA and PCR assays.

## Abbreviations

Abbreviations used are EV71: enterovirus 71; SA: sialic acid; SA-α2,3Gal: sialic acid: alpha 2,3 galactose; MOI: multiplication of index; and IFA: immunofluorescent assay.

## Competing interests

The authors declare that they have no competing interests.

## Authors' contributions

BY carried out most of the studies and drafted the manuscript. HC participated parts of the studies and art works. KDY provided grant supports and supervised the study progress and final report. All authors read and approved the final manuscript.
